# Hospital Finance

**Published:** 1922-07

**Authors:** 


### HOSPITAL FINANCE.

### THE PROGRESS OF THE COMBINED APPEAL FOR LONDON HOSPITALS.

As we go to press, more than a quarter of the £500,000 aimed at by the Hospitals of London Combined Appeal has come in, although the "intensive fortnight" (June 25-July 8) has not begun. Empire Flag Day alone produced £50,000. The King's reference, in his autograph letter to the Organising Committee, to the homecoming of the Prince of Wales and his interest in the fund of which he is President, produced an immediate increase in the number of contributions received. An anonymous donor sent £10,000 "as a welcome home gift."

Among the special features of the "intensive fortnight" are the London Hippodrome gala performance of "The Review of Revues" (June 26); the matinée at Wyndham's Theatre, organised by Lady Tree (June 27); Princess Mary's Ball at the Royal Albert Hall (June 28); and the League of Mercy Fête in the Royal Botanic Gardens (June 30). There is a growing demand from leading tradesmen, both in the central area and in the boroughs, for supplies of collecting-boxes, Combined Appeal stamps, button-badges and rosettes, all of which will be put on sale. Distribution is being effected through the mayoral and other committees. London omnibuses and L.C.C. tramcars will bear hospital-appeal posters during the fortnight, and 100 special collecting-boxes in the form of a pair of scales are to be placed for a month on Tube and Underground stations. No outstanding suggestion was received in response to the Executive Committee's offer of two prizes of £25 for new and practicable methods of getting in small money. There were 2,706 entries. Many were along lines already adopted; a high percentage urged progress with contributory schemes amongst wage-earners. The next most popular suggestion was that all London papers should be sold at 1d. extra per copy on an agreed day. It is intended that the winning proposals shall be developed for use in due course. A cheque, the result of a collection at a private dinner-party, reached the fund with the suggestion that all hosts should follow suit—an idea that, if taken up as it deserves, should bring in a substantial sum. A collection, to remain open until Christmas, is to be organised among the children of London schools of all types. It is hoped that this may produce as much as £50,000, as in this way over £22,000 was raised for the same object in 1920. The Executive Committee announces that in order that the public may inspect the inside working of the hospitals, arrangements have been made to receive visitors at the various institutions. Those desiring to inspect the wards, operating-theatres, etc., should make application in writing direct to the secretary of any hospital.

Lord Blyth, who suggested before the war that every well-established commercial enterprise should open a charity account in its ledger and place to its credit a small percentage of its yearly profits, has proposed a special appeal to commercial houses throughout the country to adopt this plan.

### PATIENTS' PAYMENTS AND THE SATURDAY FUND.

The British Hospitals Association is, we understand, discussing with the Metropolitan Hospital Saturday Fund the position of the Fund's subscribers in regard to patients' payments. It will be recalled that the Saturday Fund sent to all the London Hospitals in its form of inquiry the following question: "Are you prepared to give free treatment to the subscribers of the Hospital Saturday Fund, in return for any grant that may be awarded, leaving the subscribers to make any voluntary contributions they may desire?" The reply of certain hospitals was regarded by the Fund as a refusal, and for the present, for example, the local branch has withheld its grant of £1,362 from the Royal Northern Hospital group. Since discussion should now be taking place between the representatives of the hospitals and of the Fund, we have no desire to say anything that may hinder a friendly settlement. Indeed, the hospitals and the Fund have too long a record of co-operation and are too essential to each other for there to be more than a temporary cloud between them. The position is delicate, and it is timely to recognise how this has come about. A little history, a little reflection, often causes apparent difficulties to shrink to the mole-hills that they really are. To begin with, the hospital system is still in principle a voluntary one. It is supported by voluntary subscriptions, which are none the less voluntary because by agreement they are often collected weekly under voluntary organisations. One of these is the Hospital Saturday Fund, which, like the private subscriber, originally contributed to maintain the voluntary hospitals and not to benefit itself. True, the letter-system entitled private subscribers and collective donors to recommendations, and thus, no doubt, begot the notion that their possessors had a right to admission. But the hospitals maintained, as they still do, the principle that necessity is the sole passport to early admission. If letters are a means, they are secondary. The plan of collective subscribers is only an extension of the original principle, and the latest so-called insurance schemes expressly guard against any contract with the subscribers. But in addition to these schemes, patients who are able to pay are now invited to contribute to the cost of their maintenance, and this plan has led the collective subscribers to ask that their members may be admitted free. The general answer of the hospitals has been that necessitous cases, as hitherto, will be admitted free and that grants from the subscribing bodies can, by agreement, be used towards the cost of maintenance. There are various ways of arranging this, but the principle is clear: subscriptions are given to support a charity, and to enable those unable to subscribe to receive its benefits. Now that all, save the necessitous, are liable to be asked to contribute when in hospital, subscribers, like non-subscribers, must expect that invitation. The principle remains, though its application can be modified by arrangement. But just as a subscription confers no "right" to treatment, so it confers in itself no exemption from a hospital's rules, financial or other. If it did either, hospital treatment would be confined to subscribers, and the voluntary system and the treatment of the necessitous in the hospitals would come to an end. The major principle is not really in question. The matter at issue is how to apply the new rule of patients' payments to members of subscribing bodies. That is a matter which can safely be left to the British Hospitals Association and to the Saturday Fund to adjust between them. But because small issues are apt to cloud the principles on which voluntary hospitals and voluntary subscriptions rest, it is well to refresh our memories upon their basic relations.

### A KING'S FUND FOR THE PROVINCES.

THE familiar and hitherto abortive proposal to form in the provinces a fund similar to King Edward's Hospital Fund for London, as our readers are aware, was referred to the Council of the British Hospitals Association at the recent conference. It was referred, not to be shelved, but to be gone into, practically. The general arguments for and against were those raised when King Edward's Fund was being debated. Since this Fund has been admittedly successful, we are able to regard the arguments in its favour as triumphant, subject only to the fact that provincial conditions differ from metropolitan ones. In London, when the King's Fund was founded, hospitals were hardly co-ordinated at all. They were independent, rival bodies, needing a controlling hand to reduce their anarchic relations to order. In London, too, there was virtually no local patriotism in hospital affairs. The King's Fund provided a point of rest for the general mass of subscribers. In the provinces local feeling is strong; there is more ground for the familiar fear that a central fund might either fail or adversely affect local subscriptions. However, the tendency is still necessarily in favour of greater co-ordination, some more central control, and recent departures such as the Regional Committees of the British Hospital Association and the new Local Committees formed under the Voluntary Hospitals Commission are all in this direction. It seems safe to assume that a provincial central hospital fund on the lines now proposed will eventually prove desirable. The only practical question is if the time is ripe for its establishment. To this simple point we hope that the Council of the Association will confine its attention, since the principle may be regarded as beyond dispute.

---

Mr. Charles Manning, a working bootmaker, has left practically the whole of his savings, over £2,000, to the Royal Northern Hospital.

The idea of an Old Patients' Association occasionally means a useful source of revenue. Through that connected with the Skipton and District Hospital, Yorkshire, the institution received £150 last year.

A Children's Hospital Aid Society has been started for the benefit of the East London Hospital for Children, Shadwell. It is the first Aid Society solely in connection with the hospital. The annual subscription is 4s. 4d.

The Stratford Market Employees' Hospital Bed Fund, which was only revived last summer, in seven months raised the £200 necessary to maintain the bed named after it in Queen Mary's Hospital.

The workmen and staff of Messrs. John Brown & Company, the Sheffield armament and steel firm, have allotted £3,000 from their war relief fund to the Royal Hospital and Royal Infirmary. A bed has been endowed at each institution.

The York Corporation is anxious to purchase Acomb Hall and grounds, including a farmhouse, for a maternity hospital. It has applied for leave to borrow £12,713 for the purchase and equipment.

The Westminster City Council proposes to build a new public library on a site in Orange Street at a cost of £1,500 for the interest of the Royal Dental Hospital, and at a ground rent of £500 a year.

The Newcastle City Council has approved a scheme to acquire the City Road Industrial Schools for a maternity hospital and child welfare centre. The present arrangement is expected to suffice for 20 years.

The Bermondsey and Rotherhithe War Memorial Committee have given £9,100 to Guy's Hospital towards the endowment of a new children's ward. It is the balance of a fund collected for a children's hospital, for which sufficient funds were not raised.

The alterations to Wimbledon Hospital, lately opened by Earl Haig, have added 30 beds and brought the total to 70. Some of these are in small private wards. On the opening day, when purses were received by Lady Haig, the debt on the new buildings was £8,000.

The daily services of a nurse are obtainable from the Buxton Tearfield and District Nursing Association by working class families on payment of a penny a week. This covers all children up to the age of 16 in each family. Those over 16 who work for themselves are entitled to the same privilege on payment of an extra halfpenny a week.

A contributory pension fund has been started at the Royal Alexandra Hospital for Sick Children, Brighton. The matron and sisters have contributed £185, or half the cost. The half to be paid by the hospital will be derived from the investment of a legacy. Mr. Modera, the chairman, records that two at least of the hospital's sisters have secured it for about 20 years.

The building of the new hospital at Retford, Notts, has begun. Mr. T. Heitz Denman, chairman of the governors, presented the site; and the cost is expected to be £9,000. There will be eight beds for men, eight beds for women, six for children, four beds in two private wards, a theatre, anæsthetic and X-ray unit, an ambulance stable and a mortuary.

The Tunstall War Memorial Committee is inviting subscriptions for an endowment fund to allow it to complete the purchase of Greengates House, now available from the Wolstanton and Burslem Guardians at the price of £900. Most of the £5,000 necessary for purchase, alterations and equipment is in hand, and a fifth of this sum is needed for annual maintenance.

A hospital pageant has been held on Clapham Common, for the South London Hospital for Women. The tableaux illustrated the history of medicine and nursing and included the scene from "Nicholas Nickleby" wherein Mrs. Squeers ladles brimstone and treacle to her pupils. The pageant was organised by the Clapham and District Chamber of Commerce. It is proposed by the Hospital Saturday Committee there to arrange a pageant at Hitchin.

An intensive Hospitals Week is to be held at Birmingham in the spring of 1923. The week is intended to supplement the Hospital Guild and Hospital Sunday movements and to establish an annual street collection. Hitherto the yield from local flag days has been very disappointing, but it is hoped to raise not less than £10,000 by the Lord Mayor's new scheme. Sir Gilbert Barling and others have supported the proposal, and Mrs. H. S. Richards has been appointed honorary organiser. The committee in charge of the scheme is that of the Birmingham Hospitals Guild.

